# The role of elbow positioning on arthroscopic assessment of the long head of biceps tendon in the beach chair position

**DOI:** 10.1111/ans.17764

**Published:** 2022-05-12

**Authors:** Eugene T. Ek, Jennifer N. Flynn, Glenn N. Boyce, Gayan Padmasekara

**Affiliations:** ^1^ Melbourne Orthopaedic Group Melbourne Victoria Australia; ^2^ Department of Surgery Monash Medical Centre, Monash University Melbourne Victoria Australia

**Keywords:** arthroscopy, biceps tendinopathy, long head biceps tendon, shoulder, tendonitis

## Abstract

**Background:**

Tendinopathy of the long head of biceps (LHB) tendon is a common cause of anterior shoulder pain and dysfunction. The extra‐articular portion within the bicipital groove undergoes frequent load and friction during shoulder movements and pathology within this area is frequently missed during arthroscopic assessment.

**Methods:**

We quantified the arthroscopically assessable length of tendon within the shoulder in 14 consecutive patients undergoing subpectoral biceps tenodesis. After biceps tenotomy at the superior labrum, the tagged tendon was maximally tensioned and marked at the biceps outlet with the elbow in extension and flexion. The distance in distance between the two were measured.

**Results:**

Mean distance from the superior labral insertion of the biceps to the outlet was 16.4 ± 4.1 mm (range, 11–25). With tension on the biceps with elbow extension, the mean measurable distance was 31.3 ± 6.7 mm (range, 19–45). With elbow flexion, this increased to 39.5 ± 5.9 mm (range, 25–52). Mean increase in visible tendon length was 8.2 ± 4.3 mm (range, 5–21) (*p* = 0.002).

**Conclusion:**

Elbow flexion results in an average increase of 26.2% more extra‐articular tendon visualized at arthroscopy. Therefore, we believe that elbow flexion is a useful adjunct, especially when performed in conjunction with techniques that pull the tendon into the joint, thus allowing for more complete arthroscopic assessment of the LHB, increasing detection of symptomatic biceps tendonitis.

Level of evidence: Level IV.

## Graphical abstract

Elbow flexion results in an average increase of 26.2% more extra‐articular tendon visualized at arthroscopy. Therefore, we believe that elbow flexion is a useful adjunct, especially when performed in conjunction with techniques that pull the tendon into the joint, thus allowing for more complete arthroscopic assessment of the long head of biceps, increasing detection of symptomatic biceps tendonitis.

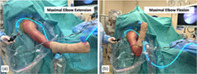

## Introduction

Tendinopathy of the long head of the biceps (LHB) tendon is a frequent cause of anterior shoulder pain and dysfunction.^1^ Patients frequently present with anterior shoulder pain associated with discomfort on forward flexion and extension and with overhead activities. Symptomatic tenosynovitis and tendinopathic changes of the LHB tendon can be present along the whole length of the tendon, from its origin at the superior glenoid tubercle and in continuity with the superior labrum, the intra‐articular portion to the extra‐articular segment located in the biciptal groove.^2^, ^3^, ^4^ This portion of the LHB tendon undergoes frequent load and friction and may contribute to classic anterior shoulder pain.^1^, ^4^ It has been reported that the distal portion of the tendon in the groove is the most common site of tendon degeneration.^5^, ^6^ It is this portion of tendon that is frequently missed by arthroscopic assessment.^3^, ^7^

The diagnosis of LHB tendinopathy is most often suspected on the basis of symptoms and clinical examination.^2^, ^3^ Ultrasound and MRI may demonstrate changes associated with the tendon such as fluid within the tendon sheath or partial tearing of the tendon.^8^ However, intraoperative arthroscopic assessment of the tendon is important in confirming the pre‐operative clinical suspicion of pathology.^3^ The biceps tendon is commonly assessed by direct visualization of the tendon for a partial tendon tear or the presence of tendonitis or tenosynovitis, evidenced by the so called ‘lipstick’ sign.^9^ This is commonly performed by pulling the extra‐articular tendon into the shoulder joint with the use of a probe or a grasper to visualize for any changes.^3^, ^10^, ^11^ Subsequent treatment includes biceps tenodesis or tenotomy, both of which have shown to be reliable options.^8^

As aforementioned, accurate visualization of the long head of biceps tendon is important when confirming the diagnosis of biceps tendinitis and determining whether or not the biceps tendon needs to be addressed surgically. During routine shoulder arthroscopy, whether in the beach chair or lateral decubitus position, a degree of traction is commonly placed on the arm with the elbow in either an extended position (lateral decubitus) or slightly flexed (beach chair). As the biceps muscle crosses both the elbow and shoulder joints, we hypothesize that with increased elbow flexion, this allows for greater excursion of the extra‐articular portion of the long head of biceps tendon into the shoulder joint, therefore allowing for greater visualization of the distal tendon.

Therefore, the purpose of this study was to perform a proof of concept study to determine the additional arthroscopically assessable length of the LHB tendon that is seen intra‐operatively when the elbow is flexed compared to extended during shoulder arthroscopy and to also document the incidence of biciptal groove tenosynovitis when assessed with this manoeuvre.

## Materials and methods

Prior to commencement of the study, ethical approval was obtained from our institution's human ethics committee (The Avenue Hospital Ethics Committee—Approval no. 207). The following method was used to arthroscopically measure the length of the long head of biceps tendon with the elbow in both extension and flexion.

### Patient position

**Fig. 1:**
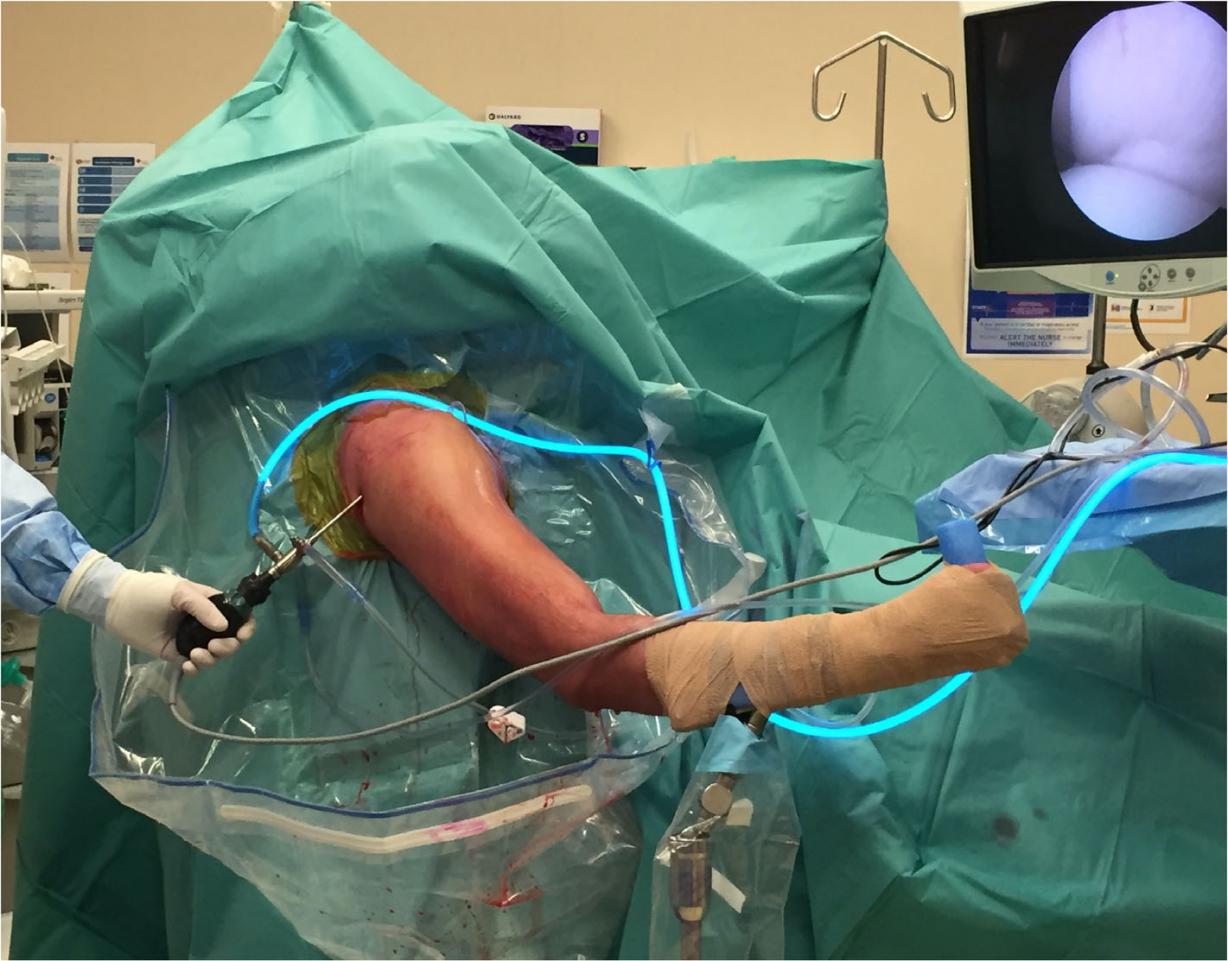
The patient is placed in a beach chair position with the head inclined approximately 70°. For the initial diagnostic arthroscopy, the operated arm is placed in a pneumatic arm holder with the shoulder at approximately 45° flexion and 30° abduction.

The patient was routinely positioned in the beach chair position with the head inclined to approximately 70° for shoulder arthroscopy. The arm was placed in a pneumatic arm holder and gentle longitudinal traction was applied with the shoulder in a standardized positioned at approximately 45° of flexion and 30° of abduction, relative to the patient's body (Fig. 1). All patients underwent either a general anaesthetic or intravenous sedation with an interscalene regional block. No patients had muscle relaxants as part of their anaesthesia.

### Tendon measurement

**Fig. 2:**
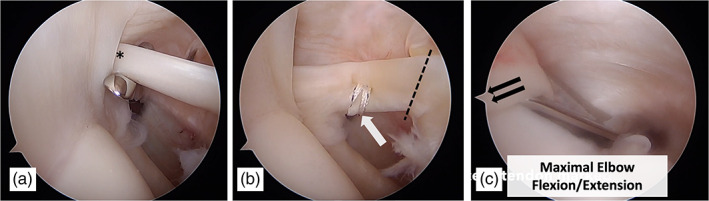
Arthroscopic view of the left shoulder from a posterior viewing portal. (a) An arthroscopic scissor is introduced through the rotator interval and a mark is made at the biceps outlet (*) with the arm in the standard diagnostic arthroscopy position. (b) A tagging suture is placed into the biceps tendon (arrow), distal to the biceps anchor for traction. The biceps tendon is tenotomized at the superior labrum (dotted line). (c) Tension is placed on the tagging suture which is coming out of the posterior portal (double arrow). The biceps tendon is then marked at the outlet with the elbow in maximal flexion and extension.

A routine diagnostic arthroscopy was then performed with the arm was placed in the standard position for diagnostic shoulder arthroscopy, which is slightly flexed at the elbow (Fig. 1). A standard posterior viewing portal was established followed by an anterolateral portal through the rotator interval where an arthroscopic scissor was passed. A mark was then made on the biceps tendon with the scissor at the level of the biceps tendon outlet (Fig. 2a).

**Fig. 3:**
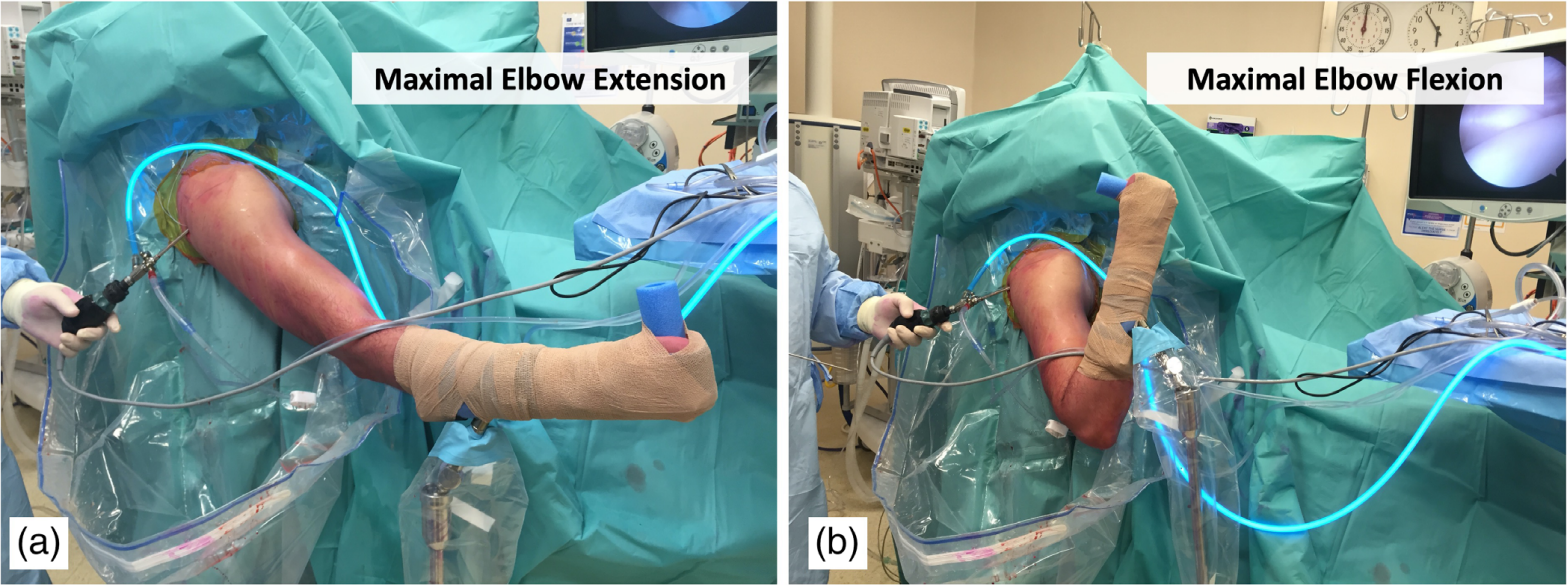
(a) The elbow is extended and positioned at approximately 20° of flexion. (b) The elbow is flexed to 130°.

The biceps tendon was then tagged proximally with a lasso suture (No. 2 Fibrewire, Arthex, Naples, FL) that was passed with an arthroscopic suture passer (Fig. 2b). The biceps tendon was then tenotomized at the level of the superior labrum and the suture holding the biceps tendon was then shuttled through to the posterior portal. Special care was made to accurately divide the biceps tendon at the junction of the superior labrum, to ensure reproducible measurements. The assistant then pulls on the suture providing maximum possible tension to the biceps tendon. The purpose of the suture was to provide tension to the biceps tendon in an anterior to posterior line of pull, in line with the biceps tendon. Maximum tension was determined when the tendon was pulled to the point where no further excursion on the tendon was observed arthroscopically. The elbow was then positioned in extension (Fig. 3a). Due to the differing lengths of the patient's arm, full extension of the elbow to 0° was not achievable with the pneumatic arm holder in all patients, hence extension of the elbow to 20° was set as the standard position (Fig. 3a). At this position, the tendon was then marked again at the level of the biceps outlet (Fig. 2c). This process was then repeated with the elbow in 130° of flexion (Fig. 3b). Of note, the position of shoulder remained unchanged, so as to not influence the position of the biceps tendon.

**Fig. 4:**
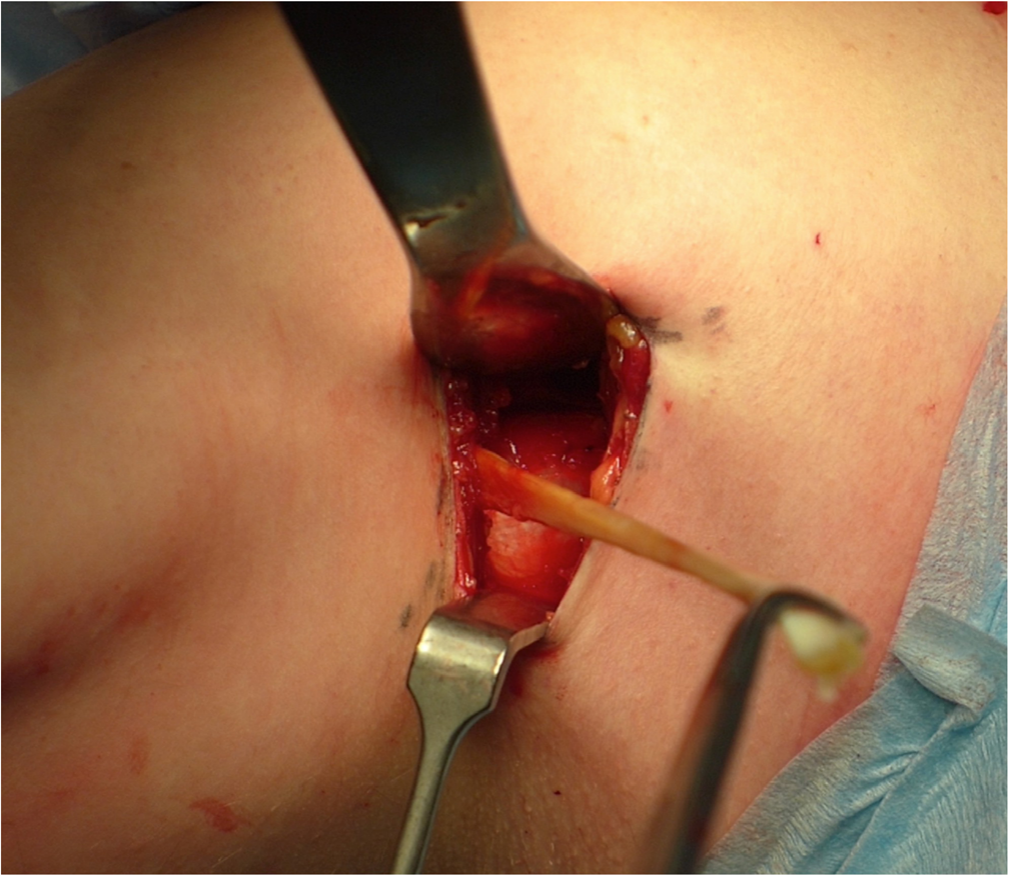
Subpectoral biceps tenodesis through an anterior axillary incision (right shoulder). The biceps is tenodesed at the musculotendinous junction and the excised tendon is retrieved for analysis.

**Fig. 5:**
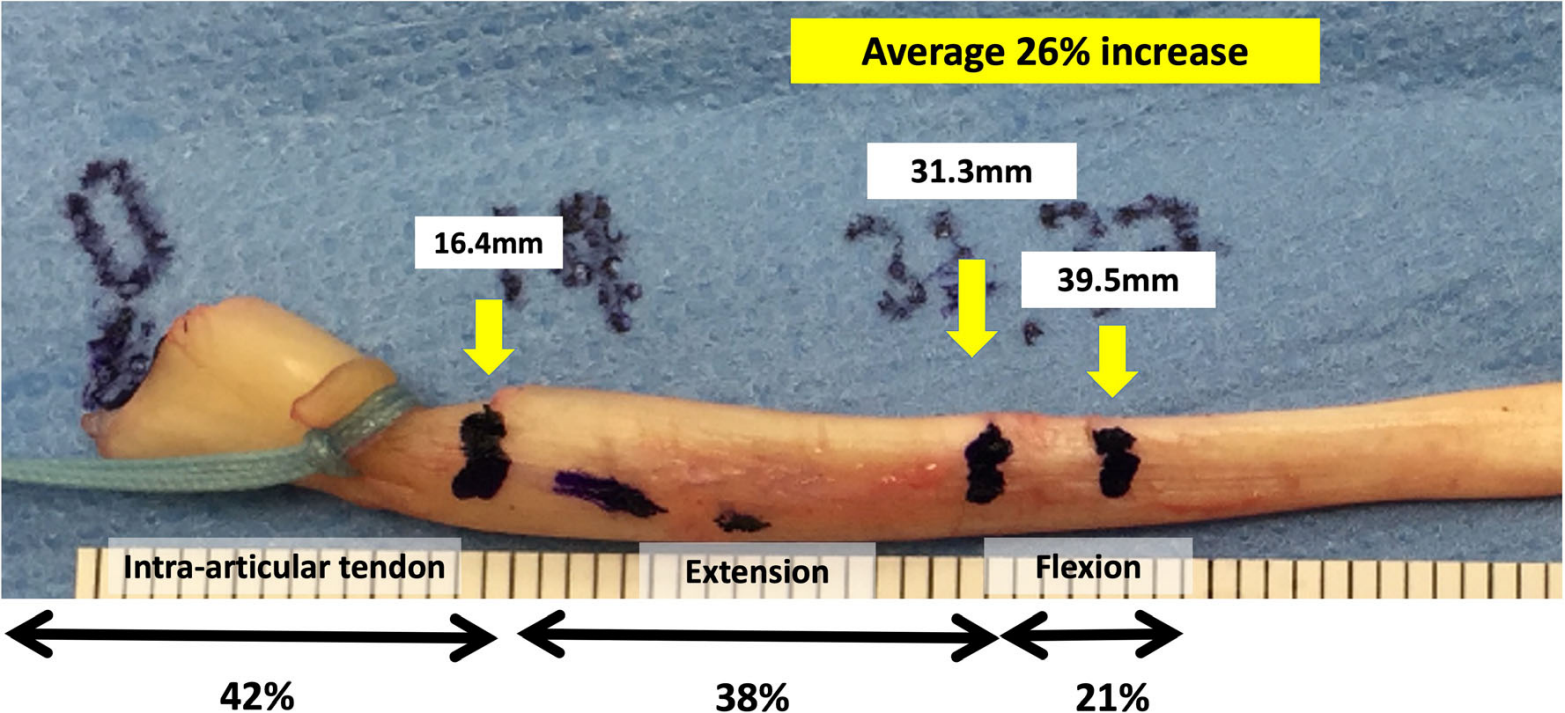
The excised long head of biceps tendon demonstrating the marks made in the tendon with the elbow at the resting position, with maximal extension and maximal flexion. Measurements are made relatively to the biceps anchor.

All patients subsequently underwent a subpectoral biceps tenodesis using unicortical button fixation (Fig. 4). As a result, part of the remaining biceps tendon was excised at the level of the musculotendinous junction. The excised tendon was then examined and the distance between the three marks were measured by a single independent examiner using a ruler with 1 mm increments (Fig. 5).

### Patients

There were 5 females and 9 males, with a total of 14 patients enrolled into this study. The mean age was 44.9 years ±11.1 (range, 30–64). This study was a consecutive, single surgeon (E.T.E) series involving patients undergoing subpectoral biceps tenodesis as part of an arthroscopic shoulder procedure. In all patients, the indication for biceps tenodesis was based on clinical features of biceps tendinopathy or superior labral pathology, such as anterior bicipital groove tenderness and a positive O'Brien's or Speeds test. This was often supported by features of LHB pathology seen on ultrasound or MRI scan. Patients were excluded if they had previous pathology of the elbow preventing full range of motion or previous surgery on the bicep tendon or extensive superior labral or intra‐articular biceps tearing preventing accurate assessment of the biceps tendon. In addition, patients who had an upper subscapularis tendon tear or a deficient anterior pulley were also excluded. The presence or absence of clear arthroscopic evidence of biceps tendonitis was documented.

### Statistical analysis

As the study was an observational study, the analysis of the data was in the form of the mean values ± standard deviations (SD) of tendon length. Mean values were compared using the Mann–Whitney *U* test for non‐parametric data. A *post hoc* analysis was performed to confirm appropriate sample size to detect a difference of greater than 20%, with an *α* = 0.05 and power of 0.8. Statistical significance was determined as a *p <* 0.05.

## Results

In the resting position, the mean arthroscopically observable distance from the superior labral insertion of the biceps to the biceps outlet was 16.4 mm ± 4.1 (range, 11–25). When the elbow was extended and maximal tension applied to the biceps tendon, the mean distance from the superior labral insertion of the biceps to the biceps outlet increased to 31.3 mm ± 6.7 (range, 19–45) (*p* < 0.0001). With elbow flexion, the mean length increased to 39.5 mm ± 5.9 (range, 25–52) (*p* < 0.0001), when compared to the resting position (Fig. 5).

Overall, there was an average increase of 8.2 mm ± 4.3 (range, 5–21) between elbow extension and flexion seen in this group, which was statistically significant (*p* = 0.002) (Table 1). This represented a mean increase of 26.2% of the observable biceps tendon. In all patients, tenosynovitis of the long head of biceps tendon was observed during arthroscopy and when placing traction on the tendon. Furthermore, tenosynovitis was seen throughout the extra‐articular portion of the long head of biceps tendon when the tendon was excised prior to subpectoral biceps tenodesis.

**Table 1:** Measurements of the excised biceps tendon demonstrating the increased visualization of the biceps tendon with elbow position

Visualized intra‐articular long head of biceps tendon	Length (mm)
At standard position	16.4 ± 4.1 (range, 11–25)
Maximal tension on biceps, elbow fully extended	31.3 ± 6.7 (range, 19–45)
Maximal tension on biceps, elbow fully flexion	39.5 ± 5.9 (range, 25–52)
Difference elbow extension to elbow flexion	8.2 ± 4.3 (range, 5–21)

*Note*: Length was measured from the biceps anchor to the mark on the biceps tendon at biceps outlet, Mean ± SD.

## Discussion

In this study we demonstrate that the extent of visualization of the extra‐articular biceps tendon can be increased with elbow flexion. As such, this would provide surgeons a better appreciation of the degree of long head of biceps tendon pathology and aid in the decision‐making process with respect to need of subsequent biceps tenotomy/tenodesis.

In clinical practice, the decision to treat the LHB is often made on the basis of preoperative clinical assessment of the patient.^12^, ^13^ While various imaging modalities, such as ultrasound and MRI can assist in the diagnosis, these are commonly associated with false negative results.^6^ Hence, intraoperative assessment of the biceps tendon during shoulder arthroscopy is a useful diagnostic adjunct, especially if features of tenosynovitis, the ‘lipstick’ sign or partial tearing of the tendon are seen.^7^, ^14^

However, a recent systematic review concluded that standard shoulder arthroscopy is associated with incomplete visualization of the extra‐articular LHB tendon, based on 575 patients and 18 cadaveric specimens included in the study.^15^ Jordan and Saithna *et al*. identified that the accuracy of various clinical test relies on arthroscopic confirmation of pathology as the gold standard, hence if evaluation of the LHB tendon is incomplete, this may lead to false negative results.^15^ Therefore, for those patients in whom the decision is based on intra‐operative assessment, any manoeuvre that allows for a more accurate view of the tendon may lead to improved outcomes if pathology is identified and addressed.^16^, ^17^

The purpose of this present study was to perform a proof of concept study with respect to elbow position and LHB tendon visualization. We used a tension suture excursion technique whereby maximal longitudinal tension was placed on the tendon through a tagging stitch. Although we did not standardize the amount of tension placed on the tendon, the tendon was maximally pulled to the point and no further excursion of the tendon was possible. This technique would most likely provide a greater degree of excursion of the biceps tendon compared to what would normally be seen by using a routine arthroscopic probe or obturator and pulling the biceps tension into the joint, which most surgeons would use in practice to view the intra‐articular tendon.

In this study, all patients were predetermined to have a biceps tenodesis, thus allowing us to tenotomize the tendon and placing longitudinal tension on it. As such, we do not advocate that this technique be part of routine arthroscopic assessment. Another manoeuvre that may also improve visualization of the extra‐articular biceps tendon would be to place the shoulder in increased flexion. However, this was not studied as we found that this was not a practical addition due to the limitations of intra‐articular viewing with the shoulder in a flexed position.

Previous studies have measured the various lengths of different portions of the long head of biceps tendon. Denard *et al*. showed that 25 mm of LBH tendon is located intra‐articularly.^18^ This observation is substantially more than that described in our current study. This may be due to the fact that that study was conducted in cadaveric specimens with the shoulder placed in the neutral adducted position, as opposed to 30° of abduction, which was used in our study. Bennett described elbow flexion and shoulder elevation for evaluating the LHB tendon at the level of the bicipital groove, but did not quantify the effect of this manoeuvre.^14^ Furthermore, Hart *et al*. examined 4 cadaveric specimens and measured 18 positions of the shoulder including elbow flexion with a two pound force applied to the biceps tendon.^17^ They showed that the greatest tendon excursion of 26.6 mm was observed with the arm in a position of neutral rotation, 30° shoulder flexion, 40° shoulder abduction and 90° of elbow flexion.^17^ However, in such a study that had been performed in cadaveric specimens, there is the potential for differences in the compliance of the biceps muscle between cadaveric tissue versus living patients. In our study, which was *in vivo* and has been performed on anaesthetised, non‐muscle relaxed patients, we attempted to replicate this similar position and place a force on the biceps tendon with the elbow in extension and in elbow flexion. Therefore, this study shows realistic quantifiable assessment of the additional assessable LHB tendon length with the simple additional manoeuvre of elbow flexion.

Bhatia *et al*. has previously described a technique of biciptal groove arthroscopy requiring a superomedial portal that allows assessment of LHB tendon assessment.^16^ Though this appears to provide adequate assessment, routine use of this technique is relatively impractical due to the need for an extra superior medial portal traversing the rotator cuff.^16^ We believe that simple elbow flexion allows for a similar assessment, although it does not further evaluate the medial and lateral biceps pulleys, which has been described by other authors including Sheean *et al* who used a 70° arthroscope through a superior portal allowing detailed visualization of 26 mm of the biciptal groove and the surrounding pulleys.^19^

In a study by Saithna *et al*. they assessed the additional length of tendon viewed in both the beach chair and lateral positions for seven forequarter amputation cadaveric specimens.^11^ They found that standard arthroscopic assessment techniques fail to demonstrate the most distal part of the biceps tendon located within the biciptal groove.^11^ To improve visualization, Favorito *et al*. described a technique of displacing the LHB tendon using a nerve hook and reported that elbow flexion was unnecessary as the patient usually has muscle relaxant as part of the anaesthetic, however no justification for this statement had been provided and there was no objective assessment of visualized LHB tendon with elbow flexion.^2^ They described an additional 3–5 cm of LHB tendon visualization utilizing a nerve hook to pull the tendon into the glenohumeral joint, which was significantly more than the 14.9 mm demonstrated in our present study. Gilmer *et al*. similarly demonstrated 30 mm of excursion of tendon into the joint utilizing an arthroscopic grasper, though no additional manoeuvre of elbow flexion was performed.^10^ Festa *et al*. also reported a 19 mm increase in excursion utilizing an arthroscopic probe.^3^ From the results of our study, we believe that the combination of the above techniques ie. using some form of instrument or grasper to pull the tendon into the joint, combined with elbow flexion, would significantly improve extra‐articular biceps tendon visualization.

There are various limitations to our study. First, as mentioned above, the technique of tenotomizing the biceps tendon and then placing traction suture that exits out of the posterior portal, is not a practical and reproducible method for routine clinical assessment of the biceps tendon, especially when the decision to address the biceps tendon is still in question. However, this is a proof of concept study to demonstrate that there is increased visualization of the biceps tendon with elbow flexion. Secondly, the while the position of the elbow flexion was easily reproduced intra‐operatively, obtaining extension of the elbow was sometime difficult due to the varying lengths of the patient's arm relative to the pneumatic arm holder. In patients with longer arms, full extension was achievable, however, in shorter arms, to obtain full extension, this required increased flexion of the shoulder. As a result, we decided to set elbow extension at 20° as this was reproducible in all patients. Another limitation of this study is that, in principle, increasing elbow flexion with the patient positioned in the lateral decubitus position may be challenging and impractical, especially for traction devices that rely on pulley weights. This could be overcome by using a pneumatic traction device that allows both longitudinal traction on the shoulder and flexion at the elbow, similar to when positioned in the beach chair position.

## Conclusion

The simple manoeuvre of elbow flexion allows a further 8.2 mm of LHB tendon to be viewed intra‐articularly, which represents approximately a 26.2% increase in observable tendon. Therefore, elbow flexion can be a useful adjunct, especially when performed in conjunction with other techniques that pull the tendon into the joint, therefore allowing for increased arthroscopic visualization of the LHB tendon and assisting in clinical decision making with respect to treatment of the biceps tendon.

## Conflict of interest

None declared.

## Author contributions

**Eugene TH Ek:** Conceptualization; investigation; methodology; project administration; supervision; writing – review and editing. **Jennifer Flynn:** Conceptualization; formal analysis; investigation; methodology. **Glenn Boyce:** Formal analysis; investigation. **Gayan Padmasekara:** Investigation; methodology; writing – original draft.
